# Prognostic implications of the expression levels of different immunoglobulin heavy chain-encoding RNAs in early breast cancer

**DOI:** 10.1038/s41523-020-0170-2

**Published:** 2020-07-06

**Authors:** Christer Larsson, Anna Ehinger, Sofia Winslow, Karin Leandersson, Marie Klintman, Ludvig Dahl, Johan Vallon-Christersson, Jari Häkkinen, Cecilia Hegardt, Jonas Manjer, Lao Saal, Lisa Rydén, Martin Malmberg, Åke Borg, Niklas Loman

**Affiliations:** 1 Translational Cancer Research, Department of Laboratory Medicine, Lund University, Lund, Sweden; 2 Oncology and Pathology, Department of Clinical Sciences Lund, Lund University, Lund, Sweden; 3 Cancer Immunology, Department of Translational Medicine, Lund University, Malmö, Sweden; 4 Surgery, Department of Clinical Sciences Malmö, Lund University, Malmö, Sweden; 5 Surgery, Department of Clinical Sciences Lund, Lund University, Lund, Sweden

**Keywords:** Prognostic markers, Breast cancer, Tumour immunology

## Abstract

The extent and composition of the immune response in a breast cancer is one important prognostic factor for the disease. The aim of the current work was to refine the analysis of the humoral component of an immune response in breast tumors by quantifying mRNA expression of different immunoglobulin classes and study their association with prognosis. We used RNA-Seq data from two local population-based breast cancer cohorts to determine the expression of *IGJ* and immunoglobulin heavy (IGH) chain-encoding RNAs. The association with prognosis was investigated and public data sets were used to corroborate the findings. Except for *IGHE* and *IGHD*, mRNAs encoding heavy chains were generally detected at substantial levels and correlated with other immune-related genes. High *IGHG1* mRNA was associated with factors related to poor prognosis such as estrogen receptor negativity, HER2 amplification, and high grade, whereas high *IGHA2* mRNA levels were primarily associated with lower age at diagnosis. High *IGHA2* and *IGJ* mRNA levels were associated with a more favorable prognosis both in univariable and multivariable Cox models. When adjusting for other prognostic factors, high *IGHG1* mRNA levels were positively associated with improved prognosis. To our knowledge, these results are the first to demonstrate that expression of individual Ig class types has prognostic implications in breast cancer.

## Introduction

Breast cancer is a heterogeneous disease, which is illustrated by differential expression of estrogen receptor (ER) and progesterone receptor (PR), occasional prevalence of *HER2* amplification, and differences in proliferation rate which together provide the basis for the classification of breast cancer in different subgroups. The subgrouping has become more elaborate with the use of global mRNA expression analysis that has led to the identification of at least five subtypes of breast cancer—basal-like, HER2-enriched, luminal A, luminal B, and normal-like tumors^1–3^. In addition, differences in the genomic stability, somatic driver mutations, and rearrangement patterns, show that different breast cancers indeed represent fundamentally differential biological subsets^4^. The heterogeneity has important implications for prognosis and for choice of adjuvant systemic therapy. For instance, patients with ER-positive tumors are advocated endocrine adjuvant therapy, whereas *HER2*-amplified tumors can be targeted with antibody-based therapy. On the other hand, for basal-like tumors that are typically negative for both ER expression and *HER2* amplification, only chemotherapy is available today.

Other factors also contribute to heterogeneity. These include the extent of immune response and presence of specific immune cells in and around the tumor. The amount of tumor-infiltrating lymphocytes^5–16^ or certain types of macrophages^17–20^, in general or when restricted to specific breast cancer subsets, have been shown to be important for the prognosis of the disease. Immune metagenes have been discovered that may be prognostic in breast cancer in general or in more limited subgroups^21–27^. Taken together, there is an abundance of studies indicating that aspects of an immune response contain prognostic information. In addition, immune checkpoint inhibition has been demonstrated to have a therapeutic potential in breast cancer, particularly in triple-negative breast cancer, along with other types of malignancies^28,29^.

One important component of an adaptive immune response is the humoral immune system whose effector molecules are constituted by antibodies. There are several classes of antibodies, including IgM, IgD, IgG1–4, IgE, and IgA1–2. An antibody is built by two identical heavy chains (immunoglobulin heavy (IGH)) and two identical light chains. The heavy chain determines the class of the antibody. During activation of the adaptive immune system B cells, which produce antibodies that recognize relevant antigens, undergo class switch by DNA recombination. Prior to class switch, the B-cell normally expresses both IgD and IgM, whereas following class switch a B-cell produces only one type of antibody of an IgM, IgG, IgE, or IgA class. Antibodies of different classes have different functions. For instance, IgAs are dimers predominantly produced in the mucosa of organs that are in contact with the exterior, such as the airways and the gastrointestinal system, but also in the lactating breast. IgE is mainly produced during parasite infections and can also be involved in an allergic reaction. IgD is a membrane bound B-cell receptor together with IgM. IgM is expressed as a pentamer early during a primary immune response and is a potent activator of the complement system. Both IgA and IgM depend on IGJ (joining chain of multimeric IgA and IgM) to assemble as a functional multimer. The role of the different Ig classes in a tumor immune response is largely unknown but they have so far been thought to be of less importance compared to T-cell-mediated immunity.

RNA-Seq methodology enables a detailed analysis of RNAs expressed in a sample. Thus, it is possible to estimate the expression of RNA encoding each class of Ig heavy chains. We have launched the Sweden Cancerome Analysis Network—Breast (SCAN-B) project^30–32^, an on-going population-based study (clinicaltrials.gov, NCT02306096) to which we invite all new breast cancer patients in Southern Sweden. If feasible, and as long as the diagnostic evaluation is not jeopardized, fresh biopsies from the primary tumors are collected and subjected to RNA-Seq analysis. Here, we have analyzed the expression of individual *IGH*s and *IGJ* from two cohorts of breast cancer with available RNA-Seq data and show that they have different associations with tumor characteristics and that at least some of them appear to have additional prognostic value, independently of the markers used in the clinic today.

## Results

### Expression of IGH mRNAs in breast cancers

**Fig. 1:**
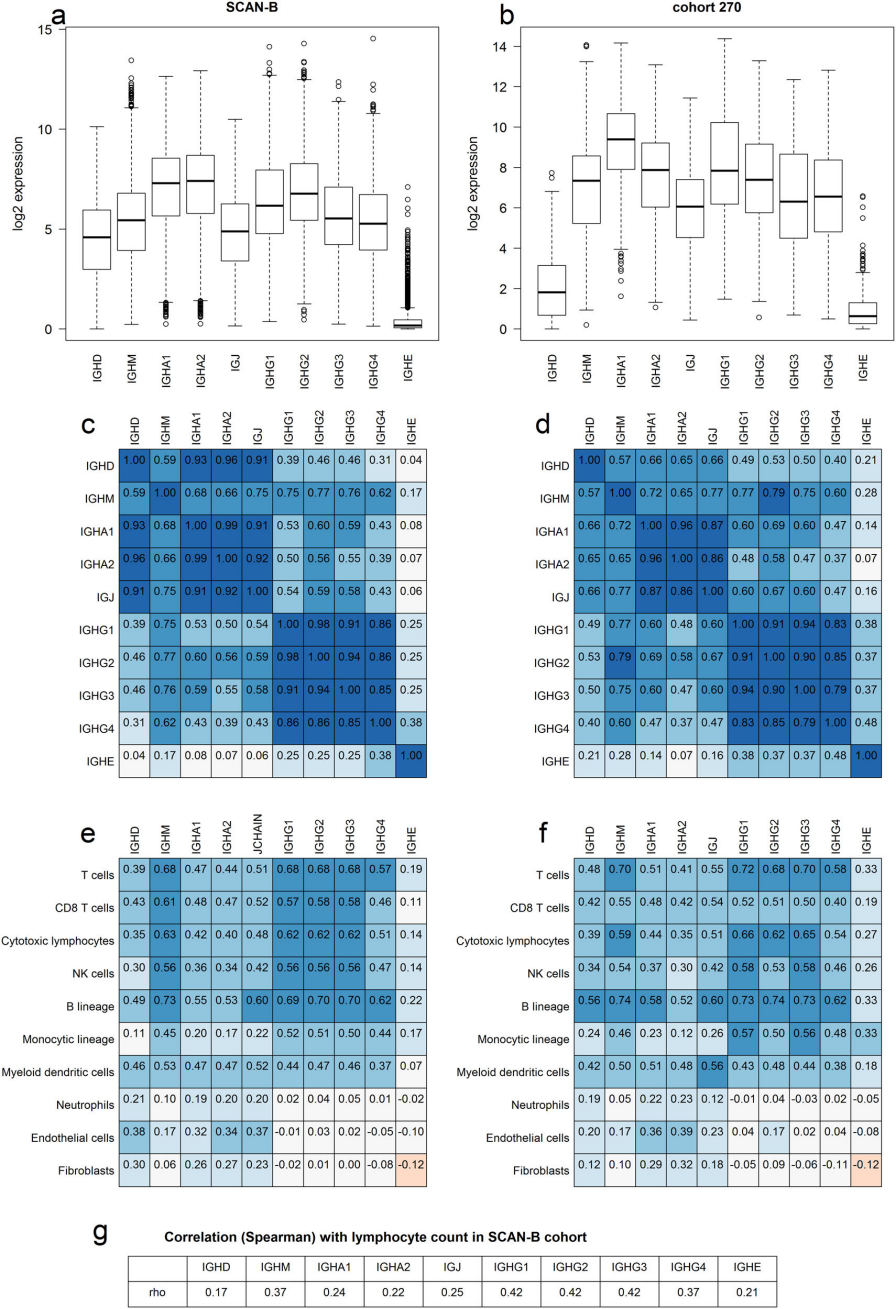
Expression and correlation of RNAs encoding IGJ and Ig heavy chains of different types. **a**, **b** Box plot of the log2 expression values of RNA encoding IGJ and the constant region of the heavy chain of different Ig classes. The data are from SCAN-B (**a**) and Cohort270 (**b**). The center line marks the median; box limits mark the upper and lower quartiles; whiskers mark 1.5× interquartile range; points mark outliers beyond this mark. Correlation matrix of the log2 expression of the indicated mRNAs in SCAN-B (**c**) and Cohort270 (**d**). The color indicates the level of the Pearson’s correlation coefficient. **e**, **f** The microenvironment cell population counter was used to obtain quantitative data for stromal cell types. Pearson’s correlation coefficient between the cell type values and log2 *IG* mRNA levels were calculated for SCAN-B (**e**) and cohort270 (**f**). The color indicates the levels of the correlation coefficient. **g** The Spearman’s correlation between indicated *IG* mRNAs and the amount of lymphocytes in the tumor, estimated on tumor section.

As a first step, the expression levels of the different Ig heavy chain and *IGJ*-encoding mRNAs were compared in the SCAN-B cohort (Fig. 1a) and in Cohort270 (Fig. 1b). Basic characteristics of the cohorts are described in Table 1 and in “Methods” section. *IGHA, IGHG*, *IGHM*, and *IGJ* mRNAs were all found at substantial levels in most tumors whereas numbers for *IGHE* mRNAs were lower, being essentially undetectable in some tumors. To analyze whether the *IGH* mRNAs are coexpressed correlation matrices were generated (Fig. 1c, d). They demonstrated that all *IGHG*s were coexpressed to a large degree. *IGHA1* and *IGHA2* mRNA levels were highly correlated and the individual *IGHG* mRNAs were also highly correlated with each other. However, the correlation between *IGHG*s and *IGHA*s were less prominent indicating that these Ig classes are not always coexpressed in a tumor. *IGJ* was highly correlated to both *IGHM* and the *IGHA*s, which is in line with *IGJ* encoding the joining chain necessary for the production of functional IgA dimers and IgM pentamers.

**Table 1:** Basic characteristics of Cohort270 and the SCAN-B cohort.

Cohort	Cohort270		SCAN-B	
Age at diagnosis (years)	Median = 62 (29–92)		Median = 64 (24–96)	
Node status	Positive	113 (43%)	Positive	1167 (37%)
	Negative	152 (57%)	Negative	2011 (63%)
	Missing	5	Missing	93
Tumor size	≤20 mm	135 (50%)	≤20 mm	2119 (65%)
	>20 mm	134 (50%)	>20 mm	1118 (35%)
	Missing	1	Missing	34
Estrogen receptor	Positive (≥10%)	233 (86%)	Positive (≥10%)	2831 (92%)
	Negative (<10%)	37 (14%)	Negative (<10%)	240 (8%)
			Missing	200
Progesterone receptor	Positive (≥10%)	205 (76%)	Positive (≥10%)	2552 (87%)
	Negative (<10%)	65 (24%)	Negative (<10%)	382 (13%)
			Missing	333
HER2 amplification	Not amplified	231 (86%)	Not amplified	2729 (87%)
	Amplified	39 (14%)	Amplified	420 (13%)
			Missing	122
Grade	Grade = 1	33 (12%)	Grade = 1	495 (15%)
	Grade = 2	123 (46%)	Grade = 2	1532 (48%)
	Grade = 3	114 (42%)	Grade = 3	1183 (37%)
			Missing	61
Endocrine therapy	Missing		Yes	2536 (78%)
			No	714 (22%)
			Missing	21
Chemotherapy	Missing		Yes	1299 (40%)
			No	1952 (60%)
			Missing	20
Recurrences	No recurrence	246	Missing	
	Recurrence	19		
	Missing	5		
Follow-up time (days)	Median = 1840 (91–2533)			
Vital status	Alive	222	Alive	2935
	Deceased	48	Deceased	336
Follow-up time (days)	Median = 2332 (1031–2810)		Median = 1642 (59–2474)	
Year of diagnosis	2007–2010		2010–2015	
Hospital	Malmö		All Southern Swedish hospitals	

Basic characteristics of Cohort270 and the SCAN-B cohort.

We utilized the microenvironment cell population counter^33^ to obtain a score for matrix cell types and analyzed their correlation with *IGH* mRNAs (Fig. 1e, f). The B-lineage cell type was the top correlating cell type in both cohorts for all *IG* mRNA species analyzed, which is in line with the mRNAs being derived from B cells. The *IGHG* mRNAs were more correlated with metagenes related to T cells, cytotoxic lymphocytes, NK cells, B-lineage, and monocytic lineage than *IGJ* and *IGHA* mRNAs, which displayed higher correlation with metagenes for neutrophils, endothelial cells, and fibroblasts than *IGHG* mRNAs. We also estimated the percentage of lymphocytes on a tissue section adjacent to the piece from which RNA was extracted for RNA-Seq analysis. *IGHG* mRNA levels showed a higher correlation with the percentage of lymphocytes than *IGHA* mRNA levels (Fig. 1g), paralleling what was found for most immune cell metagenes. Taken together, the data indicate that *IGHG* mRNAs are more associated with an immune response than *IGHA* mRNAs. For further analyses we selected *IGHG1* and *IGHA2* as representative *IGHG* and *IGHA* mRNAs.

**Fig. 2:**
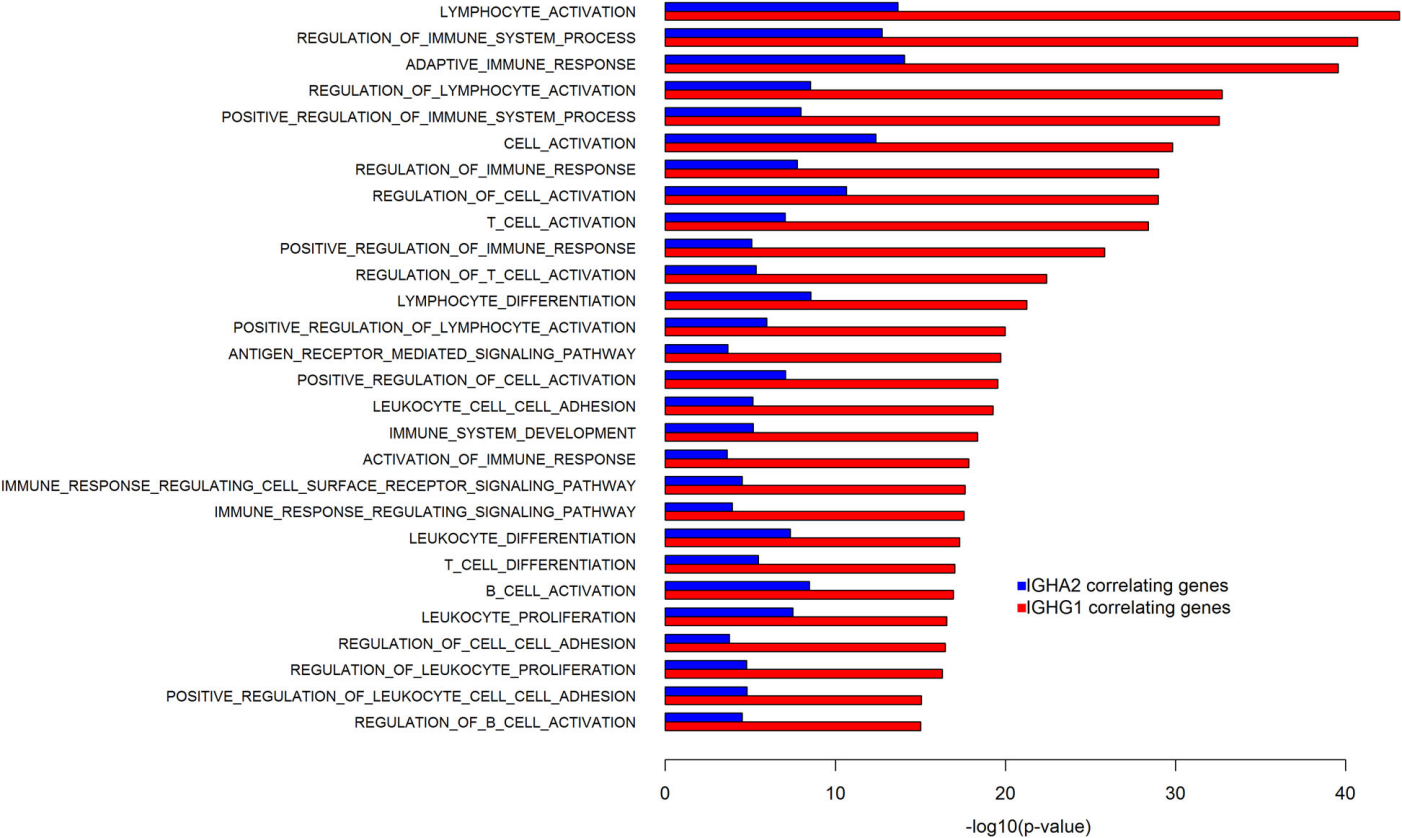
Enrichment of gene ontology sets among genes correlating with *IGHA2* and *IGHG1* mRNAs. All mRNAs in the SCAN-B cohort were analyzed for correlation with *IGHA2* and *IGHG1* mRNA expression. The top 100 correlating genes for each *IG* mRNA were selected for enrichment analysis. The enrichment was analyzed performed using the Biological Process sets defined by the Gene Ontology Consortium. Fisher’s test was used to evaluate enrichment. The graph shows the −log10 of the *p* value from the Fisher’s tests of indicated Gene Ontology Biological Process gene sets for the top 100 *IGHA2*- (blue) and *IGHG1*- (red) correlating genes. Results are shown for gene sets with a *p* value < 10^–15^ for either *IGHA2*- or *IGHG1*-correlating genes.

To take another approach to estimate the relation of IGH mRNAs with processes in the tumor we performed correlation analyses of *IGHA2* and *IGHG1* mRNAs with all other mRNAs, expressed as log2 of the FPKM, obtained with the Tuxedo RNA-Seq analysis pipeline. The top 100 correlating genes were thereafter analyzed for enrichment in Biological Process gene sets defined by the Gene Ontology Consortium (http://www.geneontology.org/), retrieved from the Molecular Signature Database (http://software.broadinstitute.org/gsea/msigdb)^34^. Sets with a *p* value < 10^–15^ for either *IGHG1*- or *IGHA2*-correlating genes were compared for enrichment (Fig. 2). Essentially all the identified sets were related to immune system processes. The data further indicate that the expression levels of *IGHG1* mRNA are more associated with an active immune response in the tumor than *IGHA2* mRNA.

### Association with clinical and pathological parameters

**Fig. 3:**
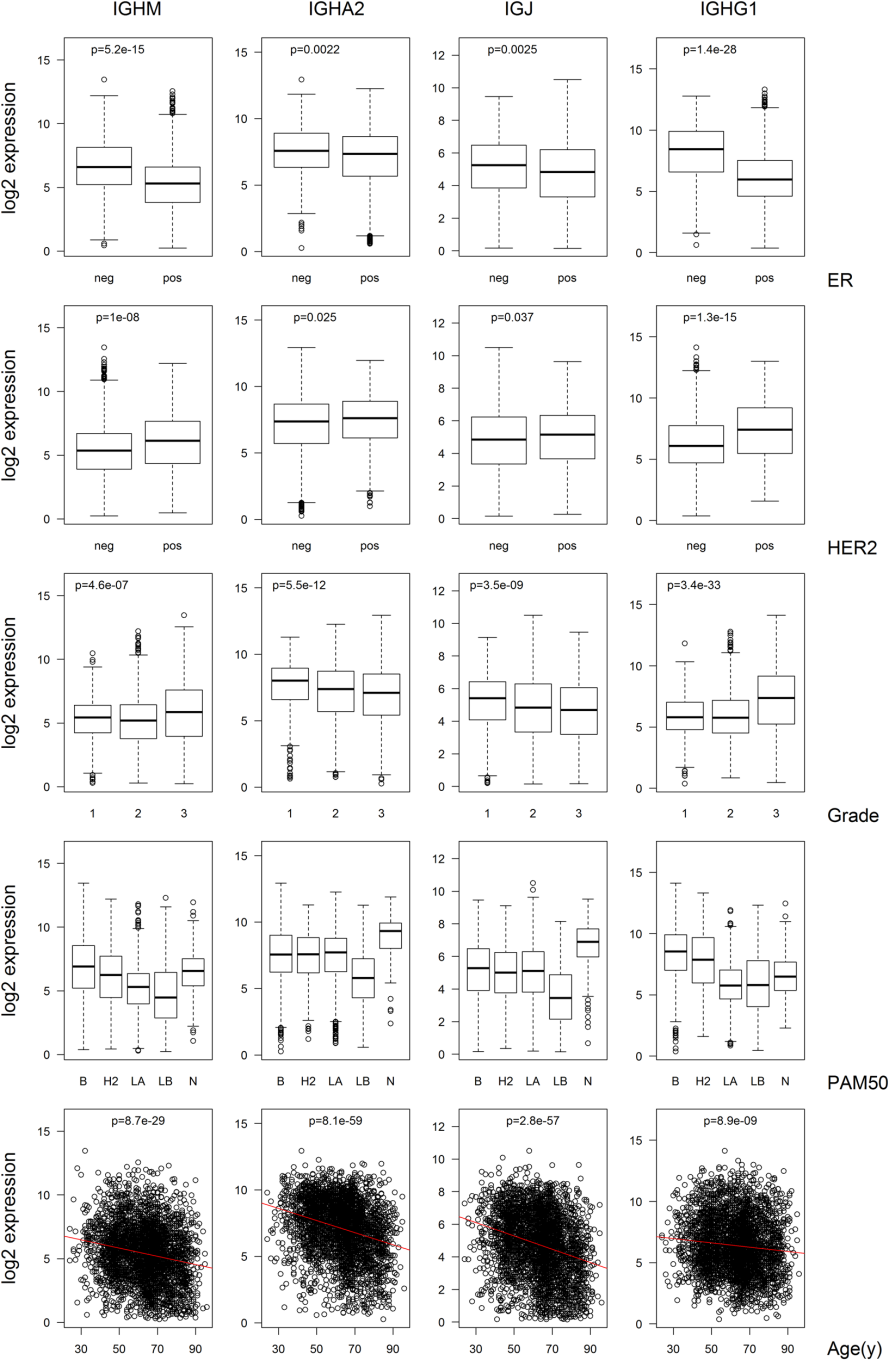
Expression of Ig heavy chain-encoding RNAs in relation to clinico-pathological parameters. Box and scatter plots of the log2 expression levels of indicated RNA species in relation to ER status, HER2 amplification, NHG grade, PAM50 subtypes, and age at diagnosis for the SCAN-B cohort. The *p* values were calculated with the *t*-test comparing positive with negative status (ER and HER2) and grade 1 versus grade 3. For age the *p* value was extracted from linear regression modeling using tlm function in R. The PAM50 subtypes are basal (B), HER2-enriched (H2), luminal A (LA), luminal B (LB), and normal-like (N). In box plots the center line marks the median; box limits mark the upper and lower quartiles; whiskers mark 1.5x interquartile range; points mark outliers beyond this mark.

The association of *IGHM*, *IGHA2*, *IGJ*, and *IGHG1* mRNA expression with established clinical parameters was analyzed in the SCAN-B cohort (Fig. 3). High expression of *IGHG1* mRNA was associated with ER negativity, HER2 amplification, and high grade. The pattern was similar but not as pronounced for *IGHM*. For *IGHA2* and *IGJ*, there was no or only a weak association with ER negativity and HER2 amplification and there was a weak association with lower grade. For molecular subtypes, *IGHA2* levels were highest in normal-like and lowest in luminal B tumors with intermediate levels in other subtypes whereas *IGHG1* expression was higher in basal and HER2-enriched tumors and lower in the other subgroups. In particular for *IGHA2* and *IGJ* expression, there was also an association with the age of the patients with levels decreasing with higher age.

### IGH mRNAs are associated with more favorable overall survival

**Fig. 4:**
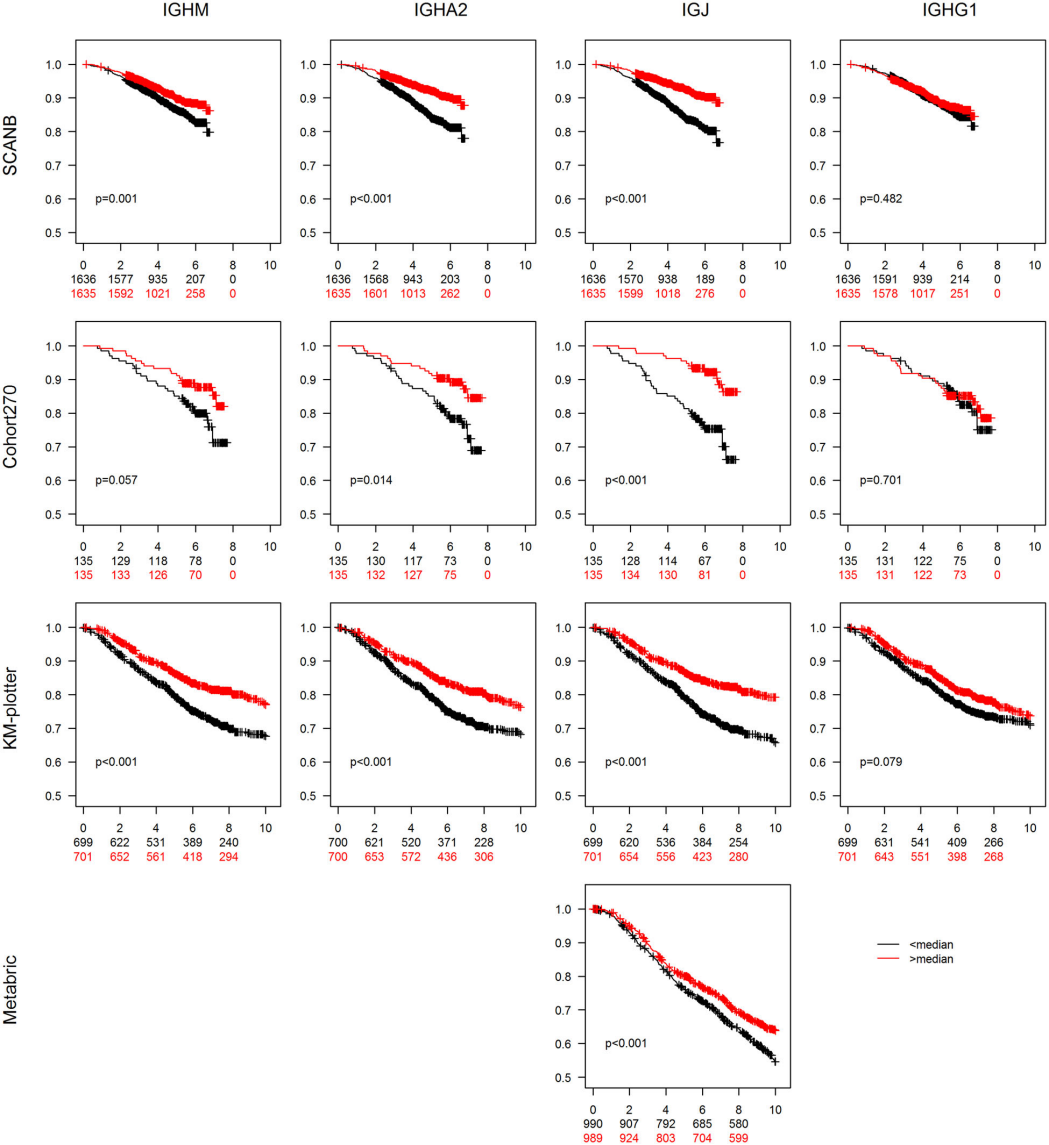
Association of IG mRNA expression with overall survival. Breast cancers from four data sets were grouped based on if the expression of *IGHM, IGHA2*, *IGJ*, and *IGHG1* mRNA was larger (red curves) or smaller (black curves) than the median expression. The figures display Kaplan–Meier curves for the indicated mRNAs (top titles) and cohorts (titles to the left) using overall survival as end point. The follow-up time on the *x*-axis is indicated in years. The *p* values were estimated with the log rank test. The *y*-axes indicate years after diagnosis and number at risk in the groups.

Different aspects of the immune profile of a tumor have been shown to correlate to prognosis. We therefore constructed Kaplan–Meier curves using overall survival as end point and dichotomized the mRNA expression on greater or smaller than the median (Fig. 4). In both the SCAN-B cohort and in Cohort270 higher levels of *IGHA2* or *IGJ* mRNA were associated with improved survival whereas this was not the case for *IGHG1*. We also utilized the Kaplan–Meier plotter^35^, which is an assembly of several breast cancer cohorts analyzed by microarray technology and thus enables analysis of a large number of breast cancer cases. Using the biomaRt package in R and the Ensembl database, probes for *IGHM*, *IGHA2*, *IGJ*, and *IGHG1* were identified and data were dichotomized on the median. The analysis showed a clear association of *IGHA2* and *IGJ* with improved survival whereas no association was seen for *IGHG1*, in line with the SCAN-B cohort and the Cohort270. *IGHM* mRNA was also associated with improved prognosis in the three cohorts but not as strongly as *IGHA2* and *IGJ*. Furthermore the Metabric cohort^36,37^ was used. Only *IGJ* of the investigated mRNAs is annotated in this data set. For *IGJ* the pattern was the same as in the other cohorts.

Since *IGH* mRNA expression was related to several known prognostic markers, a multivariable Cox modeling was performed using the SCAN-B cohort, which contains more than 3000 subjects (Table 2). We used two different models adjusting for major prognostic factors. One model was based on diagnostic parameters used in clinical routine today, while for the other one PAM50-based subgrouping was utilized. In model 1, stratification was done for patient age and chemotherapy treatment while ER status, HER2 amplification, node status, tumor size and grade were included as variables. Model 2 was stratified for PAM50 subgroup, patient age and chemotherapy while including node status and tumor size as variables. For *IGH* mRNAs, the normalized log2 expression value was used as continuous variable.

**Table 2:** Overall survival – multivariable Cox models.

Variable	IGHM HR	IGHM *p* value	IGHA2 HR	IGHA2 *p* value	IGJ HR	IGJ *p* value	IGHG1 HR	IGHG1 *p* value	Metabric IGJ HR	Metabric IGJ *p* value
**Model 1— stratified for age, chemotherapy**										
Ig gene log2 expr	0.86 *0.76–0.97*	0.014	0.86 *0.76–0.97*	0.012	0.88 *0.77–0.99*	0.041	0.86 0.76–0.97	0.015	0.93 *0.89–0.97*	<0.001
Node status pos vs neg	1.11 *0.86–1.43*	0.436	1.12 *0.86–1.45*	0.388	1.12 *0.86–1.45*	0.405	1.10 *0.85–1.43*	0.461	1.42 *1.23–1.65*	<0.001
Size >20 mm	1.74 *1.33–2.28*	<0.001	1.75 *1.34–2.28*	<0.001	1.76 *1.35–2.29*	<0.001	1.77 *1.36–2.31*	<0.001	1.36 *1.19–1.57*	<0.001
ER status pos vs neg	0.53 *0.35–0.79*	0.002	0.54 *0.36–0.81*	0.003	0.55 *0.36–0.82*	0.004	0.52 *0.34–0.79*	0.002	0.84 *0.69–1.03*	0.097
HER2 status pos vs neg	1.31 *0.89–1.91*	0.166	1.31 *0.90–1.92*	0.164	1.29 *0.88–1.89*	0.185	1.32 *0.90–1.93*	0.159	1.45 *1.18–1.78*	<0.001
Grade 3 vs 1 or 2	1.62 *1.22–2.14*	<0.001	1.54 *1.16–2.04*	0.003	1.55 *1.17–2.06*	0.002	1.67 *1.26–2.21*	<0.001	1.17 *1.01–1.35*	0.033
**Model 2—stratified for PAM50 subtype, age, chemotherapy**										
Ig gene log2 expr	0.84 *0.75–0.94*	0.003	0.85 *0.76–0.96*	0.010	0.85 *0.75–0.97*	0.008	0.85 0.76–0.96	0.008	0.96 *0.91–1.00*	0.055
Node status pos vs neg	1.49 *1.16–1.92*	0.002	1.50 *1.17–1.93*	0.002	1.50 *1.17–1.92*	0.002	1.49 *1.16–1.92*	0.002	1.52 *1.30–1.79*	<0.001
Size >20 mm	1.76 *1.37–2.27*	<0.001	1.77 *1.37–2.28*	<0.001	1.77 *1.38–2.28*	<0.001	1.79 *1.39–2.30*	<0.001	1.30 *1.12–1.51*	<0.001

Multivariable Cox proportional hazards models of SCAN-B (first eight columns) and Metabric with overall survival as end point. Two models were analyzed for each Ig mRNA. The normalized log2 Ig mRNA expression was used as continuous variable in both models. Model 1 also include node status, tumor size, ER status, HER2 status, and grade as variables and stratification was done for patient age and chemotherapy treatment. Model 2 included node status and tumor size as variables and stratification was done for PAM50 subtype, patient age, and chemotherapy treatment. Data indicate hazard ratios with 95% confidence intervals (italics) and *p*-values. The number of cases in the SCAN-B cohort was 2817 (265 events) in Model 1 and 3131 (310 events) in Model 2. For Metabric there were 1960 cases with 1128 events.

The Cox modeling revealed an independent association of *IGHA2* mRNA expression with improved prognosis (HR = 0.86, 95% CI: 0.76, 0.97 for model 1 and HR = 0.85, 95% CI: 0.76, 0.97 for model 2). The multivariable modeling demonstrated that, when adjusted for other prognostic markers, *IGHG1* mRNA was also associated with better survival (HR = 0.86, 95% CI: 0.76, 0.97 for model 1 and HR = 0.85, 95% CI: 0.76, 0.97 for model 2). Lastly, both *IGJ* and *IGHM* mRNAs were also associated with improved survival in both models. The same models were applied for *IGJ* in the Metabric cohort which revealed a similar association with prognosis in model 1 (HR = 0.93, 95% CI: 0.89, 0.97) and a tendency to an association in model 2 (HR = 0.96, 95% CI: 0.91, 1.00).

### Association of IG mRNAs with recurrence-free survival

**Fig. 5:**
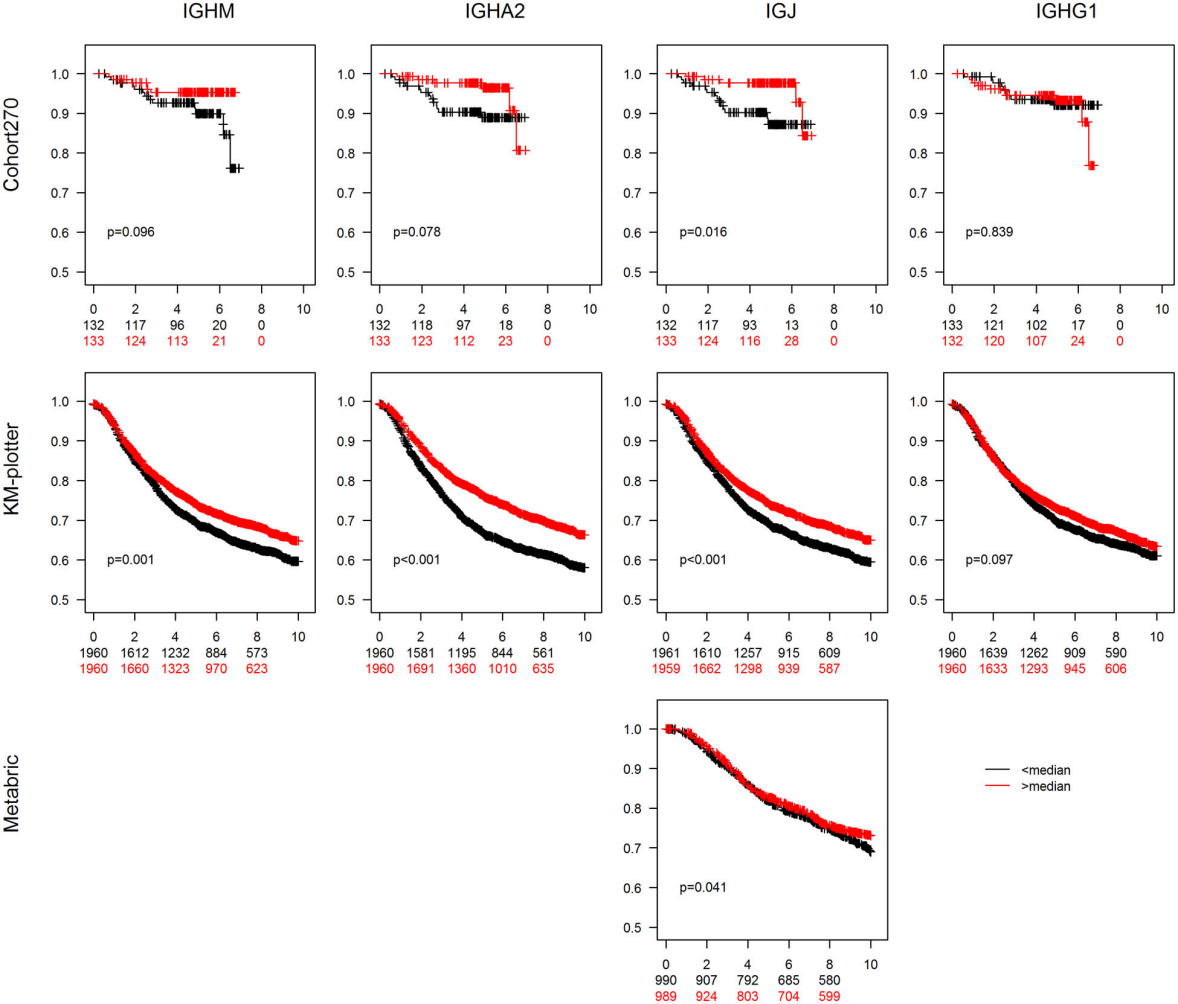
Association of IG mRNA expression with recurrence-free survival. Breast cancers from three data sets were grouped based on if the expression of *IGHM, IGHA2*, *IGJ*, and *IGHG1* mRNA was larger (red curves) or smaller (black curves) than the median expression. The figures display Kaplan–Meier curves for the indicated mRNAs (top titles) and cohorts (titles to the left) using recurrence-free survival as end point. The follow-up time on the *x*-axis is indicated in years. The *p* values were estimated with the log rank test. The *y*-axes indicate years after diagnosis and number at risk in the groups.

We next analyzed the association of the mRNA expression levels with recurrence-free survival for the cohorts where these data are available (Fig. 5). *IGJ* mRNA was associated with improved outcome in all data sets. For *IGHA2* and *IGHM* mRNA the pattern was the same in the KM-plotter data set and a nonsignificant tendency to an association was seen in the Cohort270. *IGHG1* mRNA expression was, as for overall survival, not associated with recurrence-free survival.

There are too few subjects in Cohort270 for multivariable modeling and there are no data on recurrence in the SCAN-B cohort. We therefore performed Cox modeling with the Cohort270 stratifying for each of the factors age, node status, tumor size, ER status, HER2 status, and grade separately (Table 3). *IGJ* mRNA was associated with improved survival in all models and the same was true for *IGHA2* mRNA, except when stratifying for grade where the *p* value was 0.098. *IGHG1* mRNA was not associated with prognosis in any of the models.

**Table 3:** Recurrence free survival – stratified models.

Strata	IGHM HR	IGHM *p* value	IGHA2 HR	IGHA2 *p* value	IGJ HR	IGJ *p* value	IGHG1 HR	IGHG1 *p* value
Age	0.70 *0.43–1.16*	0.168	0.60 *0.38–0.94*	0.026	0.53 *0.33–0.85*	0.009	1.04 *0.64–1.71*	0.868
Node status	0.61 *0.37–1.00*	0.052	0.57 *0.37–0.88*	0.012	0.47 *0.30–0.76*	0.002	0.94 *0.58–1.54*	0.819
Size	0.71 *0.46–1.10*	0.128	0.66 *0.44–0.99*	0.043	0.58 *0.38–0.89*	0.012	1.04 *0.66–1.62*	0.875
ER status	0.59 *0.37–0.94*	0.027	0.59 *0.39–0.90*	0.013	0.50 *0.33–0.77*	0.002	0.91 *0.56–1.49*	0.720
HER2 status	0.67 *0.42–1.06*	0.085	0.62 *0.41–0.94*	0.023	0.54 *0.35–0.83*	0.005	1.08 *0.68–1.73*	0.741
Grade	0.66 *0.43–1.01*	0.054	0.70 *0.46–1.07*	0.098	0.56 *0.37–0.85*	0.006	0.82 0.53–1.26	0.370

Univariable Cox proportional hazards models of the cohort270 with recurrence-free survival as end point. The modeling was done with the normalized log2 of the expression of indicated Ig mRNA and each model was stratified for variable indicated in the rightmost column. Data indicate hazard ratios with 95% confidence intervals (italics) and *p* values.

### Analysis of breast cancer subgroups

The size of the SCAN-B cohort allows for analysis of breast cancer subgroups (Table 4). The analyses were done both in subgroups defined by ER and PR positivity and HER2 amplification as well as those defined by the PAM50 subtypes. Cox modeling was done in the subgroups adjusting for node status, tumor size, age of the patient, and chemotherapy treatment. Both *IGHA2* and *IGJ* mRNA expression were positively associated with prognosis in the partially overlapping triple-negative (HR = 0.47, 95% CI: 0.27, 0.77 for *IGHA2* and HR = 0.58, 95% CI: 0.39, 0.93 for *IGJ*) and basal-like (HR = 0.68, 95% CI: 0.52, 0.88 for *IGHA2* and HR = 0.69, 95% CI: 0.51, 0.94 for *IGJ*) breast cancers, whereas neither *IGHM* nor *IGHG1* mRNA was significantly associated with prognosis in these groups. However, there was a tendency to an association of *IGHG1* mRNA and given the limited size of the cohort a prognostic value of *IGHG1* mRNA in these subgroups cannot be excluded. In the ER-positive HER2-negative subgroups none of the *IGH* mRNAs were significantly associated with prognosis but if the analysis was restricted to patients with age at diagnosis below the median all four (*IGHM, IGHA2, IGJ*, and *IGHG1*) mRNAs were associated with improved prognosis. In the generally ER-positive Luminal A and B subtypes the association was not significant with the exception for *IGHG1* in Luminal A cancers restricted to patients with age at diagnosis below 65 years (HR: 0.50, 95% CI: 0.26, 0.96).

**Table 4:** Overall survival – multivariable Cox models of breast cancer subgroups.

Subset	IGHM HR	IGHM *p* value	IGHA2 HR	IGHA2 *p* value	IGJ HR	IGJ *p* value	IGHG1 HR	IGHG1 *p* value
**ER/HER2 subgroups, models adjusted for grade, size, node status, age, chemotherapy**								
ER− PR− HER2−, *n* = 133, events = 24	0.74 *0.50–1.10*	0.141	0.47 *0.29–0.77*	0.002	0.58 *0.36–0.93*	0.025	0.68 *0.44–1.05*	0.081
ER+ HER2−, *n* = 2324, events = 202	0.88 *0.76–1.02*	0.089	0.91 *0.79–1.05*	0.189	0.92 *0.79–1.06*	0.241	0.89 *0.77–1.03*	0.120
ER+ HER2−, age < 66, *n* = 1213, events = 53	0.72 *0.53–0.96*	0.026	0.72 *0.55–0.95*	0.018	0.70 *0.53–0.93*	0.015	0.71 *0.52–0.96*	0.026
HER2+, *n* = 382, events = 46	0.83 *0.60–1.15*	0.261	0.79 *0.55–1.14*	0.205	0.85 *0.59–1.23*	0.396	0.81 *0.60–1.11*	0.189
**PAM50 subgroups, models adjusted for size, node status, age, chemotherapy**								
Basal, *n* = 314, events = 62	0.79 *0.61–1.02*	0.066	0.68 *0.52–0.88*	0.003	0.69 *0.51–0.94*	0.019	0.78 *0.60–1.02*	0.074
HER2 enriched, *n* = 298, events = 48	0.80 *0.60–1.06*	0.116	0.72 *0.53–0.98*	0.036	0.76 *0.55–1.05*	0.097	0.76 *0.57–1.00*	0.049
Luminal A, *n* = 1597, events = 110	0.83 *0.66–1.04*	0.112	0.95 *0.77–1.17*	0.620	0.92 *0.75–1.13*	0.416	0.83 *0.65–1.05*	0.119
Luminal A, age<65, *n* = 798, events = 19	0.58 *0.31–1.08*	0.086	0.62 *0.36–1.08*	0.090	0.61 *0.36–1.04*	0.070	0.50 *0.26–0.96*	0.037
Luminal B, *n* = 706, events = 76	0.93 *0.76–1.13*	0.459	0.91 *0.71–1.15*	0.424	0.87 *0.67–1.14*	0.316	0.89 *0.72–1.10*	0.293
Luminal B, age < 66, n = 373, events = 21	0.77 *0.51–1.16*	0.210	0.74 *0.46–1.17*	0.195	0.70 *0.42–1.17*	0.175	0.91 *0.60–1.36*	0.636

Multivariable Cox proportional hazards models in breast cancer subgroups of the SCAN-B cohort. The hazard ratio with 95% confidence interval in italics and *p* value for the normalized log2 expression of indicated Ig mRNAs in each subgroup are shown. Overall survival was used as end point. The subgroups were based on either parameters used in clinical diagnostics today, such as ER and HER2 status, or the PAM50 subgroups as indicated. All models were adjusted for tumor size, lymph node status, patient age, and chemotherapy treatment. Models for subgroups based on hormone receptor and HER2 status were also adjusted for grade.

### Association of IGHA2 mRNA with prognosis in relation to immune cell metagenes

**Fig. 6:**
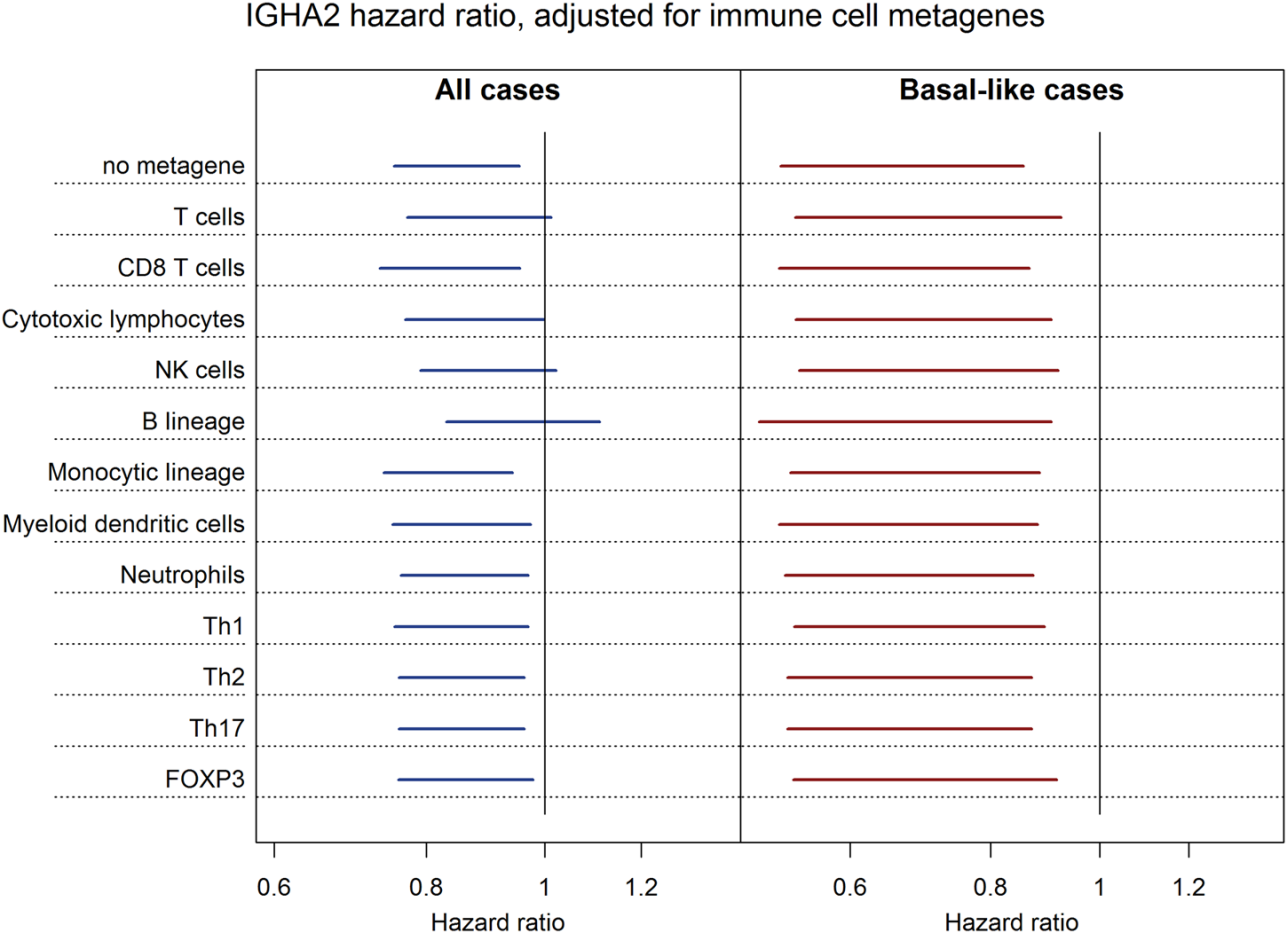
Association of IGHA2 mRNA with prognosis upon adjustment for immune cell metagenes. Multivariable Cox proportional hazards models of all cases and limited to basal-like case were performed for the SCAN-B cohort adjusting for different immune metagenes. The metagenes, indicated to the left in the figure, were generated using the microenvironment cell population Counter as for Fig. 1, as the mean log2 expression of mRNA-encoding cytokines specific for Th cells^38^, or utilizing the log2 expression of *FOXP3*. The models were also adjusted for tumor size, lymph node status, patient age and chemotherapy treatment. For the model using all cases, adjustment was also done for PAM50 subgroup. The hazards ratios with 95% confidence interval for normalized log2 *IGHA2* mRNA expression are shown in the figure.

The *IGHA* mRNA levels were not as strongly associated with immune cell metagenes as most other Ig heavy chain species (Fig. 1). The relationship between *IGHA2* mRNA levels and these metagenes was therefore examined using Cox modeling of the SCAN-B cohort (Fig. 6). In addition, we included metagenes encoding cytokines representative of Th1, Th2, and Th17 cells^38^ as well as *FOXP3* which is a marker for regulatory T cells. The models were also adjusted for PAM50 subgroup, tumor size, node status, patient age, and chemotherapy treatment. In models with all cases there was a higher hazard ratio of *IGHA2* upon adjustment of some immune cell metagenes. The hazard ratio remained below 1 but the 95% confidence interval crossed 1 for the T-cell, NK-cell, and B-lineage metagenes. The shift was most evident for the B-lineage which also was the metagene that showed the highest correlation with *IGHA2* mRNA expression. However, when the analysis was restricted to basal-like tumors there were only marginal effects on the *IGHA2* hazard ratio upon adjustment for the immune metagenes.

## Discussion

Here, we demonstrate an association of the amount of mRNAs encoding different Ig classes in the primary tumor with breast cancer prognosis. The association is primarily seen for IgA2- and IgJ-encoding RNAs, but the data indicate that upon adjustment for other prognostic factors there is a prognostic association also for IgG1. The pattern, in particular for IgA2 and IgJ, is seen in several data sets.

It is increasingly apparent that the immune response in a tumor modulates the aggressiveness of the cancer. While there are numerous reports on the importance of the cellular adaptive immune response, such as T cells, in breast cancer^8,9^ less is known about the humoral branch although the number of B-lymphocytes in a tumor has been indicated to have prognostic information^7^. RNA-Seq methodology enables detailed information of which RNAs that are expressed in a sample. Thereby it is possible to analyze which classes of immunoglobulins that are synthesized in a tumor and to what extent.

Which Ig class that a B-cell will produce is determined during the activation of an adaptive humoral immune response. The cytokine environment is generally believed to steer the class switch of a B-cell. IgAs are mainly associated with mucosal tissue and released to external lumina such as the lung or the intestine and considered to exert a first line defense against pathogens in these locations. IgA is also the major immunoglobulin in breast milk. A substantial expression of IgA mRNAs in mammary tissue would therefore be expected.

*IGHA2* expression was not as much correlated to indicators of an immune response as was the case for *IGHG* mRNAs. Instead we found that *IGHA2* mRNA in tumors decreases with the age of the patient, a pattern that was much less pronounced for *IGHG1* and *IGHM*. This may reflect a regression of active mammary tissue that conceivably comes with higher age. High levels of *IGHA2* mRNAs would then be found in tumors that resemble active and mature mammary tissue. An association of *IGHA2* expression with prognosis may therefore be related both to an association with more mature mammary tissue and with an active immune response.

The association of *IGHA2* with overall survival was found in three different data sets and in a fourth—Metabric—the same pattern was seen for *IGJ*, the expression of which correlates with that of *IGHA2*. It was also independent of other prognostic factors in essentially all models, further highlighting that it may provide added information regarding breast cancer prognosis.

The association of *IGHA2* expression with prognosis was strong in basal-like and triple-negative cancers. For these cancers tumor-infiltrating lymphocytes, and in particular CD8-positive cells, have been linked to improved prognosis^14,39,40^. The *IGHA2* mRNA levels correlate with metagenes for these cell types, which may imply that the markers represent the same characteristics in the tumor. However, for this subtype the association of IGHA2 with prognosis was largely independent of immune cell metagene levels. Furthermore, *IGHG1* mRNA levels, which were not as strongly associated with prognosis in basal-like cancers, display a higher correlation with immune cell metagenes than *IGHA2* mRNA. The association of *IGHA2* mRNA with improved prognosis may to some degree be related both to a higher immune activity in the tumor but, considering its lower correlation with immune metagenes and its independence of them in basal-like tumors in association with prognosis, other *IGHA2*-associated features are conceivably of importance.

Contrasting *IGHA2* mRNA, *IGHG1* was associated with features linked to poor prognosis, such as ER negativity, HER2 amplification, and higher grade, which is in line with what has been reported for B-lymphocytes in breast cancer tissue^7^. *IGHG1* mRNA levels were also more correlated with metagenes associated with an immune response which makes it conceivable that the IgG production, more than IgA production, reflects an active immune response in a tumor.

Given the association of *IGHG1* with factors linked to poor prognosis it would be expected that there is no association with higher *IGHG1* levels and favorable prognosis. However, using the SCAN-B cohort for which a multivariable analysis was possible, adjustment for other known prognostic factors demonstrated an association of higher *IGHG1* levels with improved prognosis. IgG is more associated with cellular immunity than other Ig species. *IGHG* mRNAs also displayed the highest correlation with metagenes for cells of the adaptive immune response. The *IGHG1* association with prognosis may therefore conceivably represent an active immune response. Thus, the data indicate that an active immune response is associated with poor prognostic factor, but when adjusting for these it is associated with improved prognosis. This is in line with reports that a B-cell signature, when adjusted for IL-8 expression is prognostic in triple-negative breast cancers^41^, that a B-cell signature is associated with good prognosis particularly in cancers with high proliferation rate^42^ or in basal-like and HER2-enriched tumors^43^, and that the amount of B-lymphocytes is associated with improved prognosis particularly in grade 3 and ER-negative tumors^7^. It suggests that, when other prognostic factors are equal, an active immune response is beneficial for the outcome.

There are some limitations to the study. These include that the results rely on only one major method, RNA-Seq, and the relatively short follow-up time which provides for a limited number of events, particularly when analyzing subgroups. More analyses are planned for and needed to deeper understand the implications of the differences in mRNA levels detected here. In particular it would be of large interest to gain further insight in the clonality and specificity of the antibodies that the mRNAs encode and whether they primarily represent antitumoral antibodies or natural antibodies or if they reflect the milk-producing function of the mammary gland. Therefore, analyses addressing the specificity of the antibodies encoded by the mRNA would significantly benefit the understanding of the association of IGH mRNAs and clinical data.

Differences in association with prognosis and/or prognostic factors between *IGHA*- and *IGHG*-encoding mRNAs have recently been observed in malignant melanoma^44^. In that context *IGHA* was associated with poor prognosis whereas higher levels of *IGHG* were associated with more favorable outcome. It was suggested that this may reflect the immune modulators secreted by IgA-producing cells which could suppress an immune response. Breast cancer is not as sensitive to immune modulators as melanoma which may be one reason for the differences in *IGHA* association with prognosis.

Our data highlight IGH-encoding mRNAs as potentially important prognostic markers in early breast cancer. We also observe differential expression of different Ig-subtypes in different breast cancer subtypes and report a potential difference in the prognostic value of the expression of the IgG and IgA classes. The use of B cells or immunoglobulin production as prognostic markers in breast cancer may benefit from a refined analysis taking into account which antibody classes that are produced in a tumor.

## Methods

### Cohorts

#### Cohort270

From September 2007 all breast cancer patients operated at Skåne University Hospital in Malmö were asked for consent for molecular analyses of their tumor. Fresh pieces from the primary tumors were stored at −80 °C prior to RNA extraction and RNA-Seq analysis^30^. For tumors which the pathologist deemed it impossible to remove a piece without jeopardizing the diagnostic work no piece was taken. From patients operated between September 2007 and the middle of 2010 270 tumors were identified for which the RNA-Seq analysis was of sufficient quality and for which the patient had no previous history of cancer, had not been subjected to neoadjuvant treatment or had no incidence of tumor in the contralateral breast. The obtaining of clinical and pathological data has been described^31^. Briefly, ER status, HER2 status, and Nottingham Histological Grade were scored by three pathologists. Other clinical data were retrieved from the Swedish Breast Cancer Register. Overall survival and recurrence, defined as local, regional, or distant metastasis, were used as end point. The data were retrieved from medical records. The molecular and some clinical data of this cohort (Cohort270) are stored at GEO (GSE81538).

#### SCAN-B cohort

Starting September 2010 all newly diagnosed breast cancer patients in the South Swedish Health Care Region were asked to participate in the SCAN-B project^30–32^ (clinicaltrials.gov, NCT02306096, tumor collection starting date August 2010, estimated ending August 2031), which largely uses the same procedures as described for Cohort270 above. From patients operated between late 2010 and early 2015, 3271 cases were identified that were primary breast cancer without diagnosis of disseminated disease. For this population-based cohort (SCAN-B) overall survival was used as end point. Clinical data were obtained from the Swedish Breast Cancer Register and overall survival data from the Swedish Population Register^31^. The molecular and clinical data are stored at GEO (GSE96058).

The two cohorts are summarized in Table 1. All included patients gave informed written consent. All patients were treated according to regional treatment guidelines that had been defined according to national and international treatment recommendations. The studies have been approved by the Lund Regional Ethical Review Board (Dnr 2007/155, 2009/658, 2010/383, 2012/58, and 2013/459).

### Tissue handling, RNA extraction, and RNA sequencing

Following surgery the specimen was transported on ice to the pathology unit, where a piece was excised from the tumor and immediately put in −80 °C (Cohort270) or RNAlater (SCAN-B). This was only done when the excision with certainty would not influence the diagnostic work. Thus, too small tumors and tumors with too diffuse borders were excluded which also ensures that the excised piece only contains material from within the tumor. RNA was extracted using the AllPrep DNA/RNA Kit (Qiagen) and sequencing libraries were prepared using 1 µg RNA as a target amount. The libraries were sequenced on an IlluminaHiSeq2000 or NextSeq500 and the reads were processed with an implemented Tuxedo pipeline utilizing Bowtie2^45^, Tophat2^46^, Cufflinks^47,48^, and hg19 reference genome annotation as described^30^.

### Calculation of IGH expression levels

Using the biomaRt^49^ package in R, chromosome positions of exons within the *IGH* locus were retrieved from Ensembl. Reads aligned to the locus were thereafter retrieved from BAM files using the RSamtools and GenomicRanges^50^ packages in R. Only reads with mapping quality above 10 were used for subsequent analysis. Based on the CIGAR parameter the number of bases from a read that had been aligned to an exon was allocated to the specific exon. The number of bases allocated to each exon for the same *IGH* class type was summed and divided by the sum of the length of the exons (in bases) and the number of reads from the library that had been aligned to the genome. The number was multiplied with 10^6^ and following addition of 1 the value was log2-transformed. We have thus obtained a number representing the log2 of counts per base per million reads. The same procedure was done for the IGJ locus as an internal control to compare the expression estimate from our method with the Tuxedo pipeline expression estimate. The IGJ locus is useful as a control since it is included as a target transcript in our Tuxedo pipeline, whereas the IGH class is not. An almost perfect correlation (*R* = 0.99) was obtained for the expression levels of IGJ using our method and the log2 FPKM obtained with the Tuxedo pipeline.

### Tumor composition

For the SCAN-B cohort the piece of the tumor that was used for RNA extraction was also used for construction of a tissue microarray. For each tumor a pathologist analyzes a hematoxylin–eosin-stained tissue microarray section for estimation of the percentage of invasive cancer, in situ cancer, fat tissue, stroma, lymphocytes, and normal breast tissue. These data were obtained for 2513 of the 3271 tumors in the cohort.

### Data analysis

All statistical analyses were done with R (version 3.6.1). Cox proportional hazards modeling was done using the coxph function. For multivariable analyses subjects with missing data were excluded. ER status (positive versus negative), HER2 amplification (positive versus negative), node positivity (>1 node versus no positive node), and tumor size (>20 versus ≤20 mm) were used as categorical values. ER and PR status were considered positive if there were more than 10% positive cells which is the standard definition for Swedish clinical practice and therefore is uniformly registered in clinical records. Grade was dichotomized (grade 3 versus grades 1 and 2). The Ig mRNA expression data was either dichotomized on the median for Kaplan–Meier curves and for analysis of association with clinical variables using Fisher or chi-squared tests or used as continuous variables in Cox proportional hazard models and in *t*-tests comparing levels between different groups. Age was binned in five year intervals. In multivariable Cox models ER status, HER2 amplification, grade, node positivity, tumor size, and Ig mRNA expression were included as variables, whereas stratification was made for age, PAM50 subgroups, chemotherapy, and cohort (Metabric).

Calculations with the microenvironment cell population counter^33^ were done using the MCPcounter package in R applying the MCPcounter.estimate function to all samples.

For gene set enrichment analysis, Gene Ontology Biological Process sets (http://www.geneontology.org/) were retrieved from the Molecular Signature Database, using version 7.0 (http://software.broadinstitute.org/gsea/msigdb)^34^. The enrichment of gene sets was analyzed with Fisher’s test.

### Reporting summary

Further information on research design is available in the Nature Research Reporting Summary linked to this article.

## Supplementary information

Reporting Summary

## Data Availability

RNA-Seq data and clinical data of cohort Cohort270 are publicly available in NCBI Gene Expression Omnibus here: https://identifiers.org/geo:GSE81538^51^. RNA-Seq data and clinical data of cohort SCAN-B, are also publicly available in NCBI Gene Expression Omnibus here: https://identifiers.org/geo:GSE96058^52^. Tumor characteristics and patient data for cohorts Cohort270 and SCAN-B, are publicly available in the figshare repository 10.6084/m9.figshare.12040326^53^. METABRIC^36,37^ data analyzed during this study, are publicly available in cBioPortal for cancer genomics here: https://identifiers.org/cbioportal:brca_metabric. For microarray sets the Kaplan–Meier plotter website (http://kmplot.com/analysis/) was used^35^. The probes used were selected using biomaRt package in R and the Ensembl database with “affy_hg_u133a” as filter. Data were downloaded as text files and the Kaplan–Meier plots were generated with R.

## Code availability

The R code for estimating IGH expression from bam files is in the figshare repository 10.6084/m9.figshare.12040326^53^.
